# A hybrid smell agent symbiosis organism search algorithm for optimal control of microgrid operations

**DOI:** 10.1371/journal.pone.0286695

**Published:** 2023-06-07

**Authors:** Salisu Mohammed, Yusuf A. Sha’aban, Ime J. Umoh, Ahmed T. Salawudeen, Sami M. Ibn Shamsah

**Affiliations:** 1 Department of Maintenance Engineering, Nigerian National Petroleum Company, KRPC Limited, Kaduna, Nigeria; 2 Department of Computer Engineering, Faculty of Engineering, Ahmadu Bello University, Zaria, Kaduna State, Nigeria; 3 Center for International Studies, Massachusetts Institute of Technology, Cambridge, Massachusetts, United States of America; 4 Department of Electrical Engineering, College of Engineering, University of Hafr Al Batin, Hafr Al Batin, Saudi Arabia; 5 Department of Electrical and Electronics Engineering, Faculty of Engineering, University of Jos, Jos, Plateau State, Nigeria; 6 Department of Mechanical Engineering, College of Engineering, University of Hafr Al Batin, Hafr Al Batin, Saudi Arabia; King Fahd University of Petroleum and Minerals, SAUDI ARABIA

## Abstract

This paper presents a hybrid Smell Agent Symbiosis Organism Search Algorithm (SASOS) for optimal control of autonomous microgrids. In microgrid operation, a single optimization algorithm often lacks the required balance between accuracy and speed to control power system parameters such as frequency and voltage effectively. The hybrid algorithm reduces the imbalance between exploitation and exploration and increases the effectiveness of control optimization in microgrids. To achieve this, various energy resource models were coordinated into a single model for optimal energy generation and distribution to loads. The optimization problem was formulated based on the network power flow and the discrete-time sampling of the constrained control parameters. The development of SASOS comprises components of Symbiotic Organism Search (SOS) and Smell Agent Optimization (SAO) codified in an optimization loop. Twenty-four standard test function benchmarks were used to evaluate the performance of the algorithm developed. The experimental analysis revealed that SASOS obtained 58.82% of the Desired Convergence Goal (DCG) in 17 of the benchmark functions. SASOS was implemented in the Microgrid Central Controller (MCC) and benchmarked alongside standard SOS and SAO optimization control strategies. The MATLAB/Simulink simulation results of the microgrid load disturbance rejection showed the viability of SASOS with an improved reduction in Total Harmonic Distortion (THD) of 19.76%, compared to the SOS, SAO, and MCC methods that have a THD reduction of 15.60%, 12.74%, and 6.04%, respectively, over the THD benchmark. Based on the results obtained, it can be concluded that SASOS demonstrates superior performance compared to other methods. This finding suggests that SASOS is a promising solution for enhancing the control system of autonomous microgrids. It was also shown to apply to other sectors of engineering optimization.

## Introduction

An autonomous microgrid generates electricity using distributed energy resources, operating as a single-entity network, without the intervention of the main grids [1]. This microgrid system coordinates different energy sources, controlled by various components, to generate and deliver stable electricity to designated load centers [2]. In Nigeria, small and medium-sized electricity consumers use microgrids as the most reliable alternative power source to the main grid system [3]. The daily activities of these consumers require intermittent switching of power load equipment to meet their varying load demands [4]. The resulting load variations induce disturbances, thereby interrupting the system frequency and voltage [5]. Effective control of steady-state frequency and voltage disruptions can be achieved using an optimized control mechanism that effectively suppresses perturbations of the line parameters between the generation sources and the distributed loads [6]. Most conventional power plants in the main grid, which rely on non-renewable resources to generate electricity, are often among the main sources of environmental pollution [7]. This is due to their high emission of greenhouse gases, leading to increased global warming [8]. Incorporation of renewable energy resources, serving as an alternative to conventional diesel generators, mitigates carbon emissions against environmental pollution [9]. Therefore, it is necessary to increase the utilization of renewable energy resources to support microgrids in power distribution systems. Solar and wind energy systems are the most common renewable energy sources readily available and easy to harness [10]. Other applications include tidal, hydroelectric, geothermal and biomass [11, 12]. However, the uncertainty of intermittent solar energy and the variability in wind speed present a challenge for hybrid energy systems [13]. High renewable utilization can be achieved by effectively optimizing renewable energy generators and control systems in autonomous microgrid operations [6, 14].

Optimization entails finding the best solution to a problem with a design objective function under constrained decision variables [15]. Modern tools for solving optimization problems include heuristic and metaheuristic algorithms. The latter is frequently employed to optimize the performance of complex dynamic systems. This is due to the intensified search for diversified solutions to obtain the best optimal solution in a reasonable time [16]. However, there is often a trade-off between exploitation and exploration when a single optimization algorithm is applied to optimize the performance of a complex system [17]. Exploitation is the fast convergence ability of an algorithm towards a suboptimal or optimal solution. Exploration is the broad navigation of an algorithm toward an optimal solution in the search space [18]. Some metaheuristic algorithms have inherently higher exploitation, while others are identified with higher exploration in the global search space. Consequently, higher exploitation often leads to premature convergence, while higher exploration causes slow convergence [19]. An optimal solution to real engineering optimization problems requires balancing exploitation and exploration [20]. Recently, metaheuristic algorithms have been modified or hybridized to optimize some engineering optimization problems effectively. A few examples of such single algorithms include Particle Swarm Optimization (PSO), Artificial Bee Colony (ABC), Genetic Algorithm (GA), Symbiotic Organisms Search (SOS) and Smell Agent Optimization (SAO) techniques [19]. Some hybridized algorithms include the Ant Colony Optimization and Genetic Algorithm (ACO-GA) [21]; Particle Swarm Optimizer and Cuckoo Search (PSO-CS) [22]; Ant Colony Optimization and Artificial Bee Colony (ACO-ABC) [23]; Seagull Optimization and Thermal Exchange (SO-TE) algorithm [24]; Improved Firefly and Symbiosis Organism Search (IF-SOS) algorithm [25]; Teaching-Learning Based Optimizer and Equilibrium Optimizer (TLBO-EO) algorithm [26]; Improved Differential Evolutionary and Neighborhood Variable Search (IDE-NVS) algorithm [27].

The PSO mimics natural interactions in fish schools or flocks of birds. It applies the random search technique in population movement using swarm intelligence to solve optimization problems [16]. In ABC optimization, the foraging behavior is applied by a bunch of honey bees searching for a food source. The bees form three cluster groups with different responsibilities. The first group is employed bees, responsible for food discovery and modification. The second group is the onlooker bees, used to obtain information from employed bees and make the best food selection. While the third group is the scouting bees, responsible for random search and defense around the hive. Each of these groups represents a candidate solution with the objective function corresponding to nectar quality for solving optimization problems [28]. In (GA), genetic creatures of individuals undergo a simulation process that reflects a degree of goodness, fitted with a potential search solution to solve optimization problems [29]. This Algorithm works on the coding of parameters such that it does not rely on the existence of real parameters. The coding process can easily handle multiple modal types of parameter optimization, which is rather challenging to handle with a classical optimization method [30]. The GA can substantially run for a reasonable number of iterations and terminates with desired chosen ending conditions such as fitness convergence when individuals meet target fitness, the maximum number of generation limits and the maximum number of stall generations [31]. In SOS, the natural interaction for survival between symbiotic organisms within an ecosystem is adapted in the search for an optimal solution to the optimization problem [32]. The algorithm applies three notable phases in the symbiotic interactions between different organisms in the ecosystem [32, 33] to solve the optimization problem. The first phase is mutualism, in which the participant species interact to benefit from each other. The second phase is commensalism, in which one of the species benefits while the other remains unhurt. The third phase is parasitism, in which one of the species benefits while the other is harmed [33]. These organisms represent the initial population, which regenerates and serve as a candidate solution to a specific problem within the ecosystem [34]. Each organism is assigned a fitness value that reflects a potential degree of goodness for the corresponding objective.

Organisms randomly interact through all three phases in repeated iterations until the desired optimization criteria are met [34]. The SOS can solve a complex optimization problem by following its dynamic approach in the search for an optimal solution [33]. The SAO is a nature-inspired algorithm which uses principles of smell molecules by an agent to track and detect the desired solution to a problem. Although SAO evaluates the Brownian motion of projected smell molecules, it also applies three interlink modes to obtain an optimal solution. These modes are sniffing, trailing, and random. In general, the modes provide an agent with a unique optimization approach and preventive measures for being trapped in local minima. Precisely, the interlinked SAO modes initiate multiple solutions, track the path, and detect the best optimal solution [19, 20, 35]. Thus, the technique mimics the static and dynamic control characteristics, regenerating multiple solutions to solve a complex problem [24] optimally. In view of the natural inspiration in metaheuristics, this paper proposes the smell agent symbiosis organisms search (SASOS) algorithm, which combines the best performance components of SAO and SOS techniques. Twenty-four subsets of multimodal benchmark test functions are used to validate the performance of the SASOS algorithm. Subsequently, the algorithm is implemented to optimize the microgrid load disturbance control model.

In the related works of hybridized algorithms, ACO-GA optimizes the cost functions, simplifying computational complexities in each optimization cycle to track the best global solution. When the algorithm was applied to solve the traveling salesman problem, it obtained the shortest optimal path compared to the other single optimization algorithms [21]. The PSO-CS hybrid solves the objective function by applying an optimal finite-time search to improve the convergence speed and accuracy toward the searchability of the best solution. The applied algorithm achieved an effective scheduling solution to the routine maintenance problem [22]. The ACO-ABC hybrid obtains initial solutions to enhance the probable solutions and discards substandard solutions using a replacement strategy. This optimizes the best chances of global solutions [23]. The SO-TE hybrid algorithm rationalizes exploitation and exploration that improves precision accuracy in the processor unit, which, in turn, improves task classification accuracy (Jia et al., 2019).

The IF-SOS hybrid algorithm was developed to improve the opposition-based learning strategy. The algorithm accelerates convergence to obtain the best global solution rapidly. Evaluation of the algorithm in benchmarks showed its effectiveness compared to single algorithms (Goldanloo & Gharehchopogh, 2022). The TLBO-EO hybrid algorithm balances exploration and exploitation at an equilibrium point in the search space. The applied algorithm optimized the gains from the Proportional-Integral (PI) controller that improved the performance of the PV system under the influence of dynamic loads. The performance results showed the viability of TLBO-EO compared to the standalone algorithms [26]. The IDE-NVS hybrid algorithm uses a two-stage optimization strategy to solve the task scheduling problem. The algorithm optimizes the allocation in different batches of scheduled tasks by minimizing the maximum completion time. When compared with the single-stage optimizers, the IDE-NVS showed a better performance [27]. Thus, the techniques mimic the static and dynamic control optimization characteristics, which regenerate multiple solutions to solve a complex problem in an optimized manner [35]. In view of the natural inspiration in metaheuristics, this paper proposes The Smell Agent Symbiosis Organisms Search Algorithm (SASOS), which combines the best performance components of SAO and SOS techniques. Twenty-four subsets of multimodal benchmark test functions are used to validate the performance of the SASOS algorithm. Subsequently, the algorithm is implemented to optimize the microgrid load disturbance control model.

In general, different related techniques have been implemented in microgrid control optimizations. These techniques can be classified into nonlinear control, robust control, optimal control, Proportional-Integral-Derivative (PID), and Model Predictive-Based Control (MPC) optimizations. For example, the PI controller was tuned for optimal control of hybrid microgrid operations using the social Spider Optimization Algorithm (SSO). The technique minimizes an absolute integral time error based on test conditions in wind invariability against modest load variations. However, with rapid load changes, the SSO tends to be trapped in local optima [1]. Similarly, an Iterative Particle Swarm Optimization (IPSO) was used to tune the PI-based fuzzy-logic control strategy. The concept improved the load frequency control performance and the power transfer quality to the loads in a hybrid microgrid. However, robust control optimization is required to handle the power network switching distortion affecting the controlled frequency’s precision [2]. In [33], the SOS optimizes frequency regulation performance at desired values in the tie-line power flow of different energy resources. Various cost functions were applied under distinctive operating conditions, such as in inter-switching between energy sources, different loading characteristics, and the impact of external disturbances on the system parameters. However, in the dynamic response of the proposed technique, there was prevailing suboptimal control and uncertainty towards the best solution in dealing with stochastic loading conditions.

In another technique, a hybrid of wavelet mutation and sine-cosine algorithms was implemented in the nonlinear Sliding Mode Controller (SMC) to improve the performance of the load frequency controller in a shipboard hybrid microgrid system. The inherent chattering characteristics of the SMC made it incapable of withstanding the adverse effects of load variations [7]. An optimal fractional-order-based MPC method was deployed in a standalone microgrid to control the frequency due to load variation. The improved performance efficacy of the developed strategy was recorded compared to the performance of conventional MPC; however, some difficulties manifested in the tuning method [8]. In another method, the H-infinity controller was deployed to regulate frequency fluctuation by optimizing linear matrix inequalities in a hybrid microgrid. The performance of the proposed controller was compared to that of the iterative PID controller; the latter exhibits better disturbance rejection and robustness in the presence of perturbations [9]. The performance of the fuzzy-logic controller was optimized using a quasi-oppositional harmony search algorithm to improve the operation of a hybrid microgrid. An optimal solution to the fluctuation of wind power was achieved using the improved scaling factors of the proposed controller.

Eventually, difficulties in the logical tuning of inference control algorithms reduce the effectiveness of control performance [10]. The model predictive centralized controller was tuned using Multi-Objective Particle Swarm Optimization (MPSO) for the power frequency control in the hybrid microgrid. The MPSO based central controller controlled the parametric variation induced by the load fluctuation via online tunning. Simulations revealed an improved robust performance and suppression of disturbances over the conventional MPC strategy. An imbalance with high exploration and slow convergence was realized in the load frequency control of this strategy [11].

Recently, the PID controller was tuned using the Smell Agent Optimization Algorithm (SAO) to regulate the frequency of interconnected multi-source renewable energy microgrids. The technique achieved superior performance in the controlled frequency under moderate load perturbations compared to the PSO and Firefly Algorithm (FA) methods of control optimization. However, under rapid step increase in loads, the SAO tuning of the controller was found to be stiff due to slow convergence [36]. Other model techniques for optimal control of hybrid microgrid operations are elucidated in [6, 12–14, 37].

In view of the related works reviewed, the hybrid algorithms played better significant roles in optimizing real engineering control problems, which are rather intricate to handle with single algorithms. Although, most of the single and hybrid algorithms are problem specific, which reduces their optimal performance in distinct applications. The proposed SASOS algorithm is aimed at providing optimal control by minimizing the imbalance between the execution speed and accuracy required in real engineering control optimization. To evaluate the efficacy of optimal control performance, SASOS is applied in the control optimization for steady-state load disturbances in microgrid operations to improve the power flow quality. This is due to the high-sensitivity responses of the power system parameters to load disturbances. Thus, the main objectives of the SASOS algorithm in microgrid control optimization are as follows. Other techniques for optimal control of hybrid microgrid operations are elucidated in [6, 12–14, 37]. In a different direction, decomposition based multiobjective algorithms were used microgrid design and sizing [38, 39].

In view of the related works reviewed, the hybrid algorithms played better significant roles in optimizing real engineering control problems, which are rather intricate to handle with single algorithms. Although, most of the single and hybrid algorithms are problem specific, which reduces their optimal performance in distinct applications. The proposed SASOS algorithm is aimed at providing optimal control by minimizing the imbalance between the execution speed and accuracy required in real engineering control optimization. To evaluate the efficacy of optimal control performance, SASOS is applied in the control optimization for steady-state load disturbances in microgrid operations to improve the power flow quality. This is due to the high-sensitivity responses of the power system parameters to load disturbances. Thus, the main objectives of the SASOS algorithm in microgrid control optimization are as follows.

- To minimize the imbalance between exploitation and exploration in the global search space.
- To improve convergence speed in optimal solutions.
- To increase the chances of achieving the desired convergence goals.
- To minimize trapping in local optima.
- To improve power quality performance in microgrid operations.
- To Minimize the effects of total harmonic disturbances on load variation control.

The rest of this paper is organized as follows: The research method that describes the literature and the fundamental reviews needed in this paper is presented in Section 2. The formulation of the microgrid control optimization problem and the development and implementation of the SASOS algorithm is detailed in the Materials and Methods Section. The results and analysis are presented in the Results Section 4. Conclusions, recommendations, and suggestions for future work are presented in the Conclusions Section.

## Materials and methods

This section first presents the fundamental components of autonomous microgrids and optimization algorithms. Second, the generalized block diagram involving the energy resources and load models is depicted and elucidated. Third, a review of the mathematical models of energy resources and optimization techniques undertaken toward optimal load disturbance control. Fourth, the microgrid components are grouped into five different categories: the renewable non-controllable (wind, solar); non-renewable controllable (diesel generator); energy storage system (battery system); the power load (synthetic load); and the optimization approach. Fifth, the mathematical transfer functions of the models are formulated. Finally, the SAO and SOS optimization techniques and the test function benchmarks are used to evaluate the SASOS algorithm.

### The mathematical modelling of autonomous microgrid

**Fig 1:**
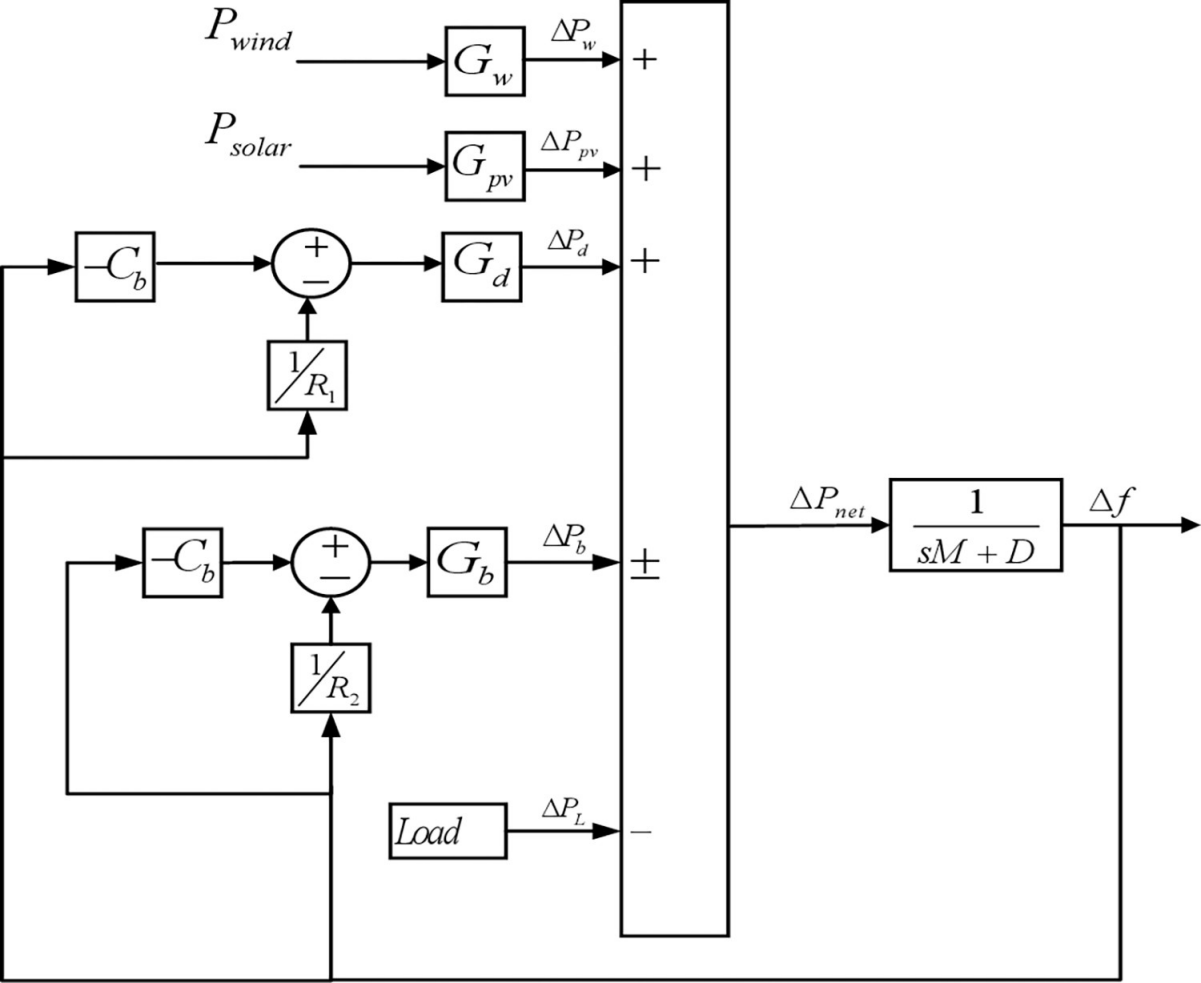
Block diagram of autonomous microgrid.

In an autonomous microgrid, various energy sources and control component models are coordinated to form a unified system model [40]. Renewable and non-renewable energy sources considered in this work include solar photovoltaics, wind turbines, and diesel generators, respectively. The approximate net power, *P*_*net*_, generated is expressed in (1) as follows [41].

$$P_{net}=P_{w}+P_{pv}+P_{dg}\pm P_{b}-P_{L}$$ (1)

where *P*_*w*_, *P*_*pv*_, and *P*_*dg*_ represent the power generated by the wind turbine, photovoltaic, and diesel generators, respectively. *P*_*b*_ represents the battery charge or discharge power, and *P*_*L*_ is the power drawn by the load. Fig 1 shows a model block diagram of interconnected energy sources and loads in an autonomous microgrid.

Fig 1 describes the interconnected energy resources and loads in an autonomous microgrid. The function blocks *G*_*w*_, *G*_*pv*_, *G*_*d*_, *G*_*b*_ and *G*_*L*_ represent the transfer function models of the wind, photovoltaics, diesel generator, battery, and load, respectively. Net changes in power output and frequency are indicated by Δ*P*_*net*_ and Δ*f*. The *M*, *D*, *R* and *C*_*b*_ represent the inertia constant, damping constant, droop characteristic, and coefficient of battery charging, respectively. A unit model of the microgrid is used to coordinate the control of individual models of energy resources [42].

#### Solar photovoltaics

Photovoltaic Cells (PV) convert the electron holes in the p-n junction into Direct Current (DC) electricity. DC electricity is converted to a suitable DC level and transformed into Alternating Current (AC) electricity with a predefined frequency and voltage. The transfer function of PV cells is given as (2).

$$G_{PV}(s)=\frac{K_{PV}}{1+\gamma_{PV}s}$$ (2)

where *K*_*PV*_ represents the transfer function gain for photovoltaics, *γ*_*PV*_ is the time constant.

#### Wind turbine

The Wind Turbine (WT) applies kinetic energy conversion to generate electricity. The propellant blades of the wind turbine rotate the prime mover directly coupled to the turbine generator that produces electricity. The transformation of the wind speed determines the amount of electricity generated into mechanical power. The expression for wind power is in (3) [41]:

$$P_{w}(s)=\frac{1}{2}C_{p}V_{w}^{3}\rho_{a}A_{w}$$ (3)

where *C*_*p*_ denotes the power coefficient, *V*_*w*_ is the wind velocity, *ρ*_*a*_ is the density of air, and *A*_*w*_ represents the area swept by the propelling blades. The transfer function dynamics of WT is expressed as follows:

$$G_{w}(s)=\frac{K_{w}}{s\gamma_{w}+1}$$ (4)

where *K*_*w*_ denotes the gain for wind turbine, and *γ*_*w*_ is the wind velocity time constant.

#### Diesel generator

The Diesel Generator (DG) is usually deployed as a non-renewable energy alternative to renewable energy resources. The DG is required to compensate for the energy shortage in renewables to meet the load demand. The transfer function for DG is expressed in (5).

$$G_{d}(s)=\frac{1}{\left(\gamma_{t}s+1)(\gamma_{g}s+1\right)}$$ (5)

where *γ*_*t*_ and *γ*_*g*_ denote the engine turbine and governor time constants, respectively.

#### Battery system

Batteries essentially preserve energy in the interconnected cells. The advantages of a battery system are high storage reliability and smoothening of renewable energy intermittency. The transfer function of the battery model is expressed in (6) as follows:

$$G_{b}(s)=\frac{K_{b}}{1+s\gamma_{b}}$$ (6)

where *K*_*b*_ denotes the battery system gain, *γ*_*b*_ is the battery time constant.

### Optimization techniques

Among the categories of optimization algorithms, metaheuristics are often used to optimize dynamic control problems [43]. This is due to their natural inspiration characteristics, soft computing, and simplicity of implementation [16]. Metaheuristics are subdivided into either swarm intelligence and evolutionary or physical creature algorithms. According to the Pareto optimal solution principle, no single optimization algorithm optimizes all constrained multi-objectives optimally but a set of trade-off solutions [34]. As such, two or more algorithms have been used to complement each other’s performance [34]. Smell Agent Optimization (SAO) and Symbiotic Organisms Search (SOS) due to the close interrelation of their natural inspiration [34].

#### Symbiotic organism search

Symbiotic Organism Search (SOS) uses natural interaction characteristics between two or more living organisms in an ecosystem [32]. These organisms exhibit survival relationships under oblige or facultative conditions. The oblige condition means that the survival of both organisms strictly depends on each other. The facultative condition means that both organisms choose mutual cohabitation in a beneficial but inessential relationship [44]. There are three distinct phases for symbiotic interrelation in SOS which are: mutualism, commensalism, and parasitism. The phases are described as follows [17, 32, 33, 45]:

1. Mutualism: in this phase, participant organisms interrelate in exchange for symbiotic benefits for survival within an ecosystem. In the mutual search space, variable vectors representing the organism *x*_*i*_ and *x*_*j*_ are deployed randomly to interact to increase survival chances. The measure of survival advantage in each organism is termed its Benefit Factor (BF). In the random interaction process, the fitness values in *x*_*i*_ and *x*_*j*_ are updated by the highest degree of survival organism known as *x*_*best*_. This leads to the generation of organisms with a new candidate solution given in (7) and (8):

$$x_{inew}^{m}=x_{i}+rand\;(∙)*(x_{best}-(\left(x_{i}+x_{j}\right)/2)*{BF}_{1})$$ (7)

$$x_{jnew}^{m}=x_{j}+rand\;(∙)*(x_{best}-(\left(x_{i}+x_{j}\right)/2)*{BF}_{2})$$ (8)

where, $x_{inew}^{m}$ and $x_{jnew}^{m}$ are new candidate solutions, *x*_*best*_ is the variable vector of the organism with the highest degree of survival, *BF*_1_ and *BF*_2_ are the benefit factors with values randomly determined between −1and 1, which indicate equal or unequal benefits between the organisms. The term (*x*_*i*_+*x*_*j*_)/2 is a mutual vector representing the organisms.

2. Commensalism: In this phase, one of the participant organisms benefits from the symbiosis relationship without affecting the other [32]. In the commensalism search space, a vector *x*_*j*_ is chosen to interact with a vector *x*_*i*_ randomly in an ecosystem such that only *x*_*i*_ benefits. The vector of the organism *x*_*j*_ does not gain or lose in the interaction [17]. The fitness value of *x*_*i*_ is updated by the organism *x*_*best*_ in a set of random numbers to generate a new candidate solution given in (9):

$$x_{inew}^{c}=x_{inew}^{m}+rand\;(∙)*(x_{best}-x_{jnew}^{m})$$ (9)

Eq (9) represents the model of the commensalism phase [17], where rand values are generated between −1 and 1 at random.

3. Parasitism: in this phase, participant organisms interact such that one benefits while the other is harmed. In the course of interaction between *x*_*i*_ and *x*_*j*_, a parasite vector $x_{i}^{p}$ is generated from random numbers to mutate *x*_*i*_. If the fitness value in the parasite vector, $x_{i}^{p}$, is better, *x*_*j*_ will be killed, and its position will be replaced by *x*_*i*_. If the fitness value immunity in *x*_*j*_ is better, $x_{i}^{p}$ will not survive in the ecosystem [33].

Recently, SOS was used to solve engineering optimization problems, such as sizing distributed generators, improving load frequency control, and minimizing power loss. This is due to its simplicity in tuning parameters and global search capability. However, the algorithm is characterized by poor exploration [33].

#### Smell agent optimization

Smell agent optimization (SAO) applies combinatorial optimization principles using the smell sensation of an agent. In pursuit of the Brownian motion of the smell molecules projected from the source, the agent tracks and detects the optimal solution to an optimization problem. For the optimal detection of these molecules, SAO applies three interlinked modes as follows [20]:

- Sniffing mode: is the degree of agent perception of the projected smell molecules from the source.
- Trailing mode: is the tracking ability of an agent to detect the projected smell molecules from the source.
- Random mode: is a distinctive search approach by an agent for optimal detection of the shortest path of projected smell molecules from the source.

The SAO is modeled based on its parameters, starting with the sniffing position given in (10) as follows [19]:

$$X_{i}^{\left(t+1\right)}=X_{i}^{\left(t\right)}+V_{i}^{\left(t+1\right)}$$ (10)

where $X_{i}^{\left(t+1\right)}$ and $X_{i}^{\left(t\right)}$ denote the current and previous positions of the molecules, respectively. $V_{i}^{\left(t\right)}$ is the velocity of the molecule. The updated sniffing position is expressed in (11).

$$X_{i}^{\left(t+1\right)}=X_{i}^{\left(t\right)}+\left(V_{i}^{\left(t\right)}+r_{0}\sqrt{\frac{3KT}{m}}\right)$$ (11)

where *r*_0_ is a random number, *m* is the mass of the molecule, *K* is the Boltzmann constant, and *T* is the temperature of the molecule [20]. The model for trailing mode is derived in (12):

$$X_{i}^{\left(t+1\right)}=X_{i}^{\left(t\right)}+r_{1}(∙)*f_{olc}*(X_{agent}^{\left(t\right)}-X_{i}^{\left(t\right)})-r_{2}(∙)*f_{olc}*(X_{worst}^{\left(t\right)}-X_{j}^{\left(t\right)})$$ (12)

where, $X_{agent}^{\left(t\right)}$ and $X_{worst}^{\left(t\right)}$ are the trailing agent and worst organism vectors, respectively. The *r*_1_(∙) and *r*_2_(∙) are randomly generated numbers at each sampling time, *f*_*olc*_ is the olfaction capacity [20].

Similarly, the random mode is formulated as in (13) [35]:

$$X_{i}^{\left(t+1\right)}=X_{i}^{\left(t\right)}+r_{3}(∙)*SM$$ (13)

where, *SM* is the constant step movement, and *r*_3_(∙) is the random penalty number [20]. The combinatorial performance of the SAO model optimizes the hybrid microgrid operations [35]. The SAO and SOS algorithms are strategically chosen in the approach to the SASOS optimization technique. While the technique applies effective smell perception as an agent in the search for a symbiosis relationship, the trailing mode of SAO introduces a single control parameter [20].

#### Performance metrics for optimization algorithms

In Computational Intelligence (CI), the performance of a newly developed, hybrid, or modified algorithm is often validated against a subset of test function benchmarks [46]. The structures of such functions are mathematical representations consisting of one or more contours [19]. The goal of any CI algorithm is to obtain the best optimal solution by minimizing or maximizing these contours under various inequality or equality constraints to satisfy the objective functions [34]. Twenty-four carefully selected multidimensional test functions in Tables 1–3, which represent a broad optimization spectrum with different degrees of complexities, are used as benchmarks in this paper.

**Table 1:** Two-dimensional benchmark test-functions.

F/No.	Function Name	D	Formula	Range	Global min
**Fn1**	Beale	2	$f(x)={\left(1.5-x_{1}+x_{1}x_{2}\right)}^{2}+{\left(2.25-x_{1}+x_{1}x_{2}^{2}\right)}^{2}+{\left(2.625-x_{1}+x_{1}x_{2}^{3}\right)}^{2}$	[−4.5,4.5]	0
**Fn2**	Easom	2	$f(x_{1},x_{2})=-\cos\left(x_{1}\right)\cos\left(x_{2}\right)exp(-{\left(x_{1}-\pi \right)}^{2}-({\left(x_{1}-\pi \right)}^{2}))$	[−100,100]	−1
**Fn3**	Matyas	2	$f(x)=0.26(x_{1}^{2}+x_{1}^{2})-0.48x_{1}x_{2}$	[−10,10]	0
**Fn4**	Bohanchevsky1	2	$f(x)=\sum_{i=1}^{n-1}\left[x_{i}^{2}+2x_{i+1}^{2}-0.3\;\cos\left(3\pi x_{i}\right)-0.4\;\cos\left(4\pi x_{i+1}\right)+0.7\right]$	[−100,100]	0
**Fn5**	Booth	2	$f(x)={\left(x_{1}+2x_{2}-7\right)}^{2}+{\left(2x_{1}+x_{2}-5\right)}^{2}$	[−10,10]	0
**Fn6**	Michalewicz2	2	$f(x)=-\sum_{i=1}^{2}\sin(x_{i}){\left(\sin\left(ix_{i}^{2}/\pi \right)\right)}^{20}$	[0,*π*]	−1.8013
**Fn7**	Schaffer	2	$f(x)\sum_{i=1}^{30}\left({\left(x_{i}^{2}+x_{i+1}^{2}\right)}^{0.25}\right)\{{\left[\sin50{\left(x_{i}^{2}+x_{i+1}^{2}\right)}^{0.1}\right]}^{2}+1\}$	[−100,100]	0
**Fn8**	Six Hump Camelback	2	$f(x)=4x_{1}^{2}-2.1x_{1}^{4}+\frac{1}{3}x_{1}^{6}+x_{1}x_{2}-4x_{2}^{2}+4x_{2}^{4}$	[−5,5]	−1.03163
**Fn9**	Bohachevsky 2	2	$f(x)=\sum_{i=1}^{n-1}\left[x_{i}^{2}+2x_{i+1}^{2}-0.3\;\cos\left(3\pi x_{i})(4\pi x_{i+1}\right)+0.3\right]$	[−100,100]	0
**Fn10**	Shubert	2	$f(x)=\left(\sum_{i=1}^{n}i\;\cos(i+1)x_{i}+i\right)\left(\sum_{i=1}^{n}i\;\cos(i+1)x_{i+1}+i\right)$	[−10,10]	−186.73

**Table 2:** Four, five, and ten-dimensional benchmark test-functions.

F/No.	Function Name	D	Formula	Range	Global min
**Fn11**	Colville	4	$f(x)=100{\left(x_{1}-x_{2}\right)}^{2}+{\left(x_{1}-1\right)}^{2}+{\left(x_{3}-1\right)}^{2}+90{\left(x_{3}^{2}-x_{4}\right)}^{2}$ $+10.1({\left(x_{2}-1\right)}^{2}+{\left(x_{4}-1\right)}^{2})+19.8(x_{2}-1)(x_{4}-1)$	[−10,10]	0
**Fn12**	Michalewicz5	5	$f(x)=-\sum_{i=1}^{2}\sin\left(x_{i}\right){\left(\sin\left(ix_{i}^{2}/\pi \right)\right)}^{20}$	[0,*π*]	−4.6877
**Fn13**	Zakharov	10	$f(x)=\sum_{i=1}^{D}x_{i}^{2}+{\left(\sum_{i=1}^{D}0.5ix_{i}\right)}^{2}+{\left(\sum_{i=1}^{D}0.5ix_{i}\right)}^{4}$	[−5,10]	0
**Fn14**	Michalewicz10	10	$f(x)=-\sum_{i=1}^{2}\sin\left(x_{i}\right){\left(\sin\left(ix_{i}^{2}/\pi \right)\right)}^{20}$	[0,*π*]	−9.6602

**Table 3:** Thirty-dimensional benchmark test-functions.

F/No	Function Name	Dimension D	Formula	Range	Global min
**Fn15**	Step	30	$f(x)=\sum_{i=1}^{n}{\left(x_{i}+0.5\right)}^{2}$	[−5.12,5.12]	0
**Fn16**	Sphere	30	$f(x)=\sum_{i=1}^{D}x_{i}^{2}$	[−100,100]	0
**Fn17**	Sum squares	30	$f(x)=\sum_{i=1}^{D}{ix}_{i}^{2}$	[−10,10]	0
**Fn18**	Quartic	30	$f(x)=\sum_{i=1}^{D}{ix}_{i}^{4}+Rand$	[−1.28,1.28]	0
**Fn19**	Schewefel 2.2	30	$f(x)=\sum_{i=1}^{D}\left|x_{i}\right|+\prod_{i=1}^{D}\left|x_{i}\right|$	[−10,10]	0
**Fn20**	Rosenbrock	30	$f(x)=\sum_{i=1}^{D-1}\left(100{\left(x_{i+1}-x_{i}^{2}\right)}^{2}+{\left(x_{i}-1\right)}^{2}\right)$	[−30,30]	0
**Fn21**	Dixon-Price	30	$f(x)={\left(x_{i}-1\right)}^{2}+\sum_{i=1}^{D}i(2x_{i}^{2}-x_{i}-1)$	[−10,10]	0
**Fn22**	Rastringin	30	$f(x)=10n+\sum_{i=1}^{n}\left[x_{i}^{2}-10\;\cos\left(2\pi x_{i}\right)\right]$	[−5.25,5.12]	0
**Fn23**	Griewank	30	$f(x)=\frac{1}{4000}-20\;\exp\left(\sum_{i=1}^{D}{\left(x_{i}-100\right)}^{2}\right)-\left(\prod_{i=1}^{D}\cos\left(\frac{x_{i}-100}{\sqrt{i}}\right)\right)+1$	[−600,600]	0
**Fn24**	Ackley	30	$f(x)=-20exp\left[-\frac{1}{5}\sqrt{\frac{1}{n}\sum_{i=1}^{D}x_{i}^{2}}\right]-exp\left[\frac{1}{n}\sum_{i=1}^{D}\cos\left(2\pi x_{i}\right)\right]+20+exp$	[−32,32]	0

Tables 1–3 consist of unimodal and multimodal test functions of different dimensions. The functions are used for SASOS validation and comparison with the SAO and SOS algorithms. The algorithms are then implemented to optimise the microgrid load disturbance control.

## The microgrid control optimization problem

This section considers the active and reactive powers that influence the system stability control in the optimization problem. The network voltage, line current, and frequency must be optimized for optimum stability to improve active and reactive load power control. The optimization problem is formulated by applying a discrete time sampling period as in [46], considering the change in active power consumed by the loads given in (14):

$$P^{k+1}={\left[T_{s}[-\frac{R}{L}P-\omega_{g}Q+\frac{3}{2L}({\left|V_{g}^{\prime }\right|}^{2}-I_{m}(V_{g}^{\prime },V_{i}^{*}))]\right]}^{k}+P^{k}$$ (14)

where *P*^*k*^ is the active power at a sample state *k*, *T*_*s*_ is the sampling time, *R*/*L* is the line resistance to inductance ratio, *ω*_*g*_ is the network frequency, *Q* is the reactive power, $V_{g}^{\prime }$ is the network voltage vector, *I*_*m*_ is the line current that the loads can draw, $V_{i}^{*}$ is the preserved energy voltage vector. Similarly, the reactive power is expressed in (15):

$$Q^{k+1}={\left[T_{s}[\omega_{g}P-\frac{R}{L}Q\pm \frac{3}{2L}I_{m}(V_{g}^{\prime },V_{i}^{*})]\right]}^{k}+Q^{k}$$ (15)

where *Q*^*k*+1^−*Q*^*k*^ indicates the power angle deviation, which at every sample time *T*_*s*_ can be represented in (16) and (17):

$$\delta {\left(V_{g}^{\prime },I_{m}\right)}^{k+1}={\left[T_{s}[\omega_{g}P-\frac{R}{L}Q\pm \frac{3}{2L}I_{m}(V_{g}^{\prime },V_{i}^{*})]\right]}^{k}$$ (16)

$$\delta (V_{g}^{\prime },I_{m})={\left[T_{s}[\omega_{g}P-\frac{R}{L}Q\pm \frac{3}{2L}I_{m}(V_{g}^{\prime },V_{i}^{*})]\right]}^{\frac{k}{k+1}}$$ (17)

The optimization problem is formulated in (18) in terms of the network-constrained parameters as follows:

$$\delta (V_{g}^{\prime },I_{m})=min\sum_{i=1}^{N-1}{\left[T_{s}[\omega_{g}P-\frac{R}{L}Q\pm \frac{3}{2L}I_{m}(V_{g}^{\prime },V_{i}^{*})]\right]}^{\frac{k}{k+1}}$$ (18)

subject to:

$$V_{gmin}^{\prime }\leq V_{g}^{\prime }\leq V_{gmax}^{\prime }$$

$$I_{m\;min}\leq I_{m}\leq I_{m\;max}$$

$$\omega_{g\;min}\leq \omega_{g}\leq \omega_{g\;max}$$

where $\delta (V_{g}^{\prime },I_{m})$ represents the power angle deviation to be minimized, *N* is the number of optimization samples, $\frac{k}{k+1}$ is the prediction time sample rate, $V_{gmin}^{\prime }$ and $V_{gmax}^{\prime }$ are the minimum and maximum network voltages, *I*_*min*_ and *I*_*max*_ are the minimum and maximum line currents that the loads can draw, *ω*_*gmin*_ and *ω*_*gmax*_ are the minimum and maximum network frequencies, respectively. The values of the synthetic variable parameters for the microgrid given in Table 4 were determined after an extensive analysis of experimental simulation.

**Table 4:** Synthetic variable parameters of autonomous microgrid system.

S/N	Parameters	Symbols	Values	S/N	Parameters	Symbols	Values
1.	Active power sensitivity	*n*_*p*_	1	10.	Weighting factor	*λ*	1
2.	Reactive power sensitivity	*n*_*q*_	0	11.	PV time constant	*γ*_*pv*_	1.8s
3.	Load active power	*P*_*L*1_	120Ω	12.	WT time constant	*γ*_*w*_	1.5s
4.	Load reactive power	*P*_*L*2_	120 + j130 Ω	13.	Battery time constant	*γ*_*b*_	0.5s
5.	Load Impedance	*Z*_*L*_	0.2 Ω	14.	Turbine time constant	*γ*_*t*_	0.4s
6.	Sampling time	*T*_*s*_	0.05s	15.	Governor time constant	*γ*_*g*_	0.08s
7.	Active droop coefficient	*K*_*p*_	0.001 rad/s/w	16.	Droop characteristics	*R*	3
8.	Reactive droop coefficient	*K*_*q*_	0.001 V/Var	17.	Inertia constant	*M*	0.1667
9.	Conversion factor	*f*_*c*_	0.1	18.	Damping constant	*D*	0.015

### Development of smell agent symbiosis organism search algorithm

The Smell Agent Symbiosis Organism Search Algorithm (SASOS) is developed based on the coding of the trailing mode of Smell Agent Optimization (SAO) with phases of Symbiotic Organism Search (SOS). The algorithm is developed to perform the optimization procedure using loops comprising mutualism phase, commensalism phase, and the introduced trailing mode. The developed step-by-step optimization procedure is shown in Algorithm 1. The initial population of the algorithm is generated using a set of random numbers. The while-loop process is developed from the mutualism phase, the commensalism phase, and the trailing mode. The process ends when the stop criteria are met.

**Algorithm 1: Smell Agent Symbiotic Organism Search**

**Input:** Initial population of organisms, create *x*_*i*_ *and x*_*j*_, *i* = 1,2,3⋯, *i*≠*j*, *set ecosize*, setup stopping criteria

**Output:** Best optimal solution

1. *iteration_number*←*iteration_number*+1

2. Identify the best organism, *x*_*best*_

3. **while** stopping criteria is not met **do**

4. **for** *i* = 1 *to ecosize* **do**

5. **Mutualism Phase**

6.  $MV=\left(x_{i}^{m}+x_{j}^{m}\right)/2$

7.  ${BF}_{1}=rand\;(.)*(1+r_{1}(0,1))$

8.  ${BF}_{2}=rand\;(.)*(1+r_{2}(0,1))$

9.   $x_{inew}^{m}=x_{i}^{m}+rand(0,1)*(x_{best}-(MV*{BF}_{1}))$

10.   $x_{jnew}^{m}=x_{j}^{m}+rand(0,1)*(x_{best}-(MV*{BF}_{2}))$

11.   Evaluate $x_{inew}^{m}$

12.   **If** $x_{inew}^{m}$ is better than $x_{i}^{m}$ **then**

13.    $x_{i}^{m}\leftarrow x_{inew}^{m}$

14.    **end if**

15.   Evaluate $x_{jnew}^{m}$

16.    **If $x_{jnew}^{m}$** is better than $x_{j}^{m}$ **then**

17.    $x_{j}^{m}\leftarrow x_{jnew}^{m}$

18.    **end if**

19. **Commensalism Phase**

20.    $x_{inew}^{c}=x_{inew}^{m}+rand(-1,1)*(x_{best}-x_{jnew}^{m})$

21. Evaluate $x_{inew}^{c}$

22.  **If** $x_{inew}^{c}$ is better than $x_{inew}^{m}$ **then**

23.    $x_{i}^{m}\leftarrow x_{inew}^{c}$

24.   **end if**

25.  **end for**

26. **Trailing Mode**

27.   *N* = 1,2,3…; number of organisms,

28.   $X_{i}^{\left(t+1\right)}=X_{i}^{\left(t\right)}+r_{1}(-1,1)*f_{olc}*(X_{agent}^{\left(t\right)}-X_{i}^{\left(t\right)})-r_{2}(-1,1)*f_{olc}*(X_{worst}^{\left(t\right)}-X_{j}^{\left(t\right)})$

29.  **Begin:**

30.  Evaluate fitness of $X_{i}^{\left(t+1\right)}$ after commensalism

31.     Obtain the best $X_{agent}^{\left(t\right)}$ and the worst $X_{worst}^{\left(t\right)}$ organisms’ positions

32.  **for** *i* = 1: *N* **do:**

33.     Determine the new position of organism using $X_{i}^{\left(t+1\right)}$

34.   **end for**

35.  Evaluate the fitness of the new position of $X_{agent}^{\left(t\right)}$

36.   **If** (new fitness position of $X_{agent}^{\left(t\right)}$ is better), **then**

37.      Accept new fitness of $X_{agent}^{\left(t\right)}$

38.   Update organisms’ position

39.   **end if**

40. **end while**

The pseudocode of SASOS in Algorithm 1 consists of SOS’s fundamental phases and the SAO’s trailing mode. Integration of the trailing mode has introduced a single control parameter into the pseudocode. Therefore, the SASOS algorithm improves performance accuracy and convergence speed and reduces the imbalance between exploration and exploitation within the global search space.

## Results and discussion

This section presents and discusses the performance of the optimization algorithm and the results obtained in microgrid simulations. SASOS, SOS, and SAO algorithms are evaluated using the 24 selected test functions. A convergence study is carried out to evaluate the effectiveness of the fitness value for each algorithm. The algorithms are implemented in their respective strategies to solve the formulated microgrid control optimization problem.

### Convergence analysis of algorithms

**Fig 2:**
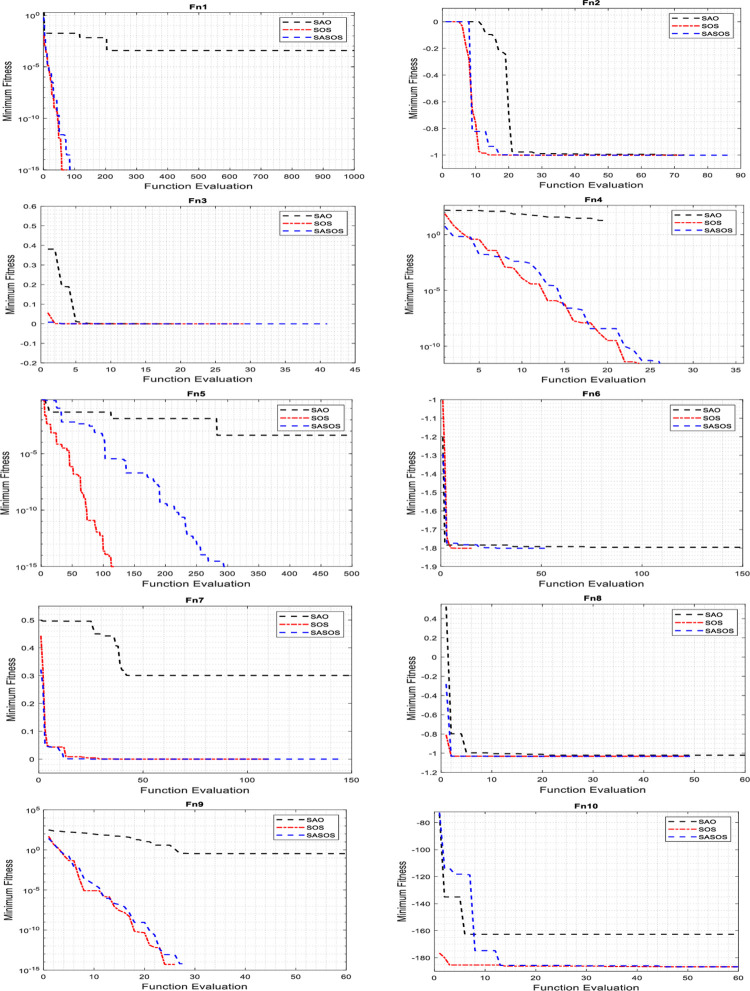
Plots of convergence curves on 2-D benchmark functions.

**Fig 3:**
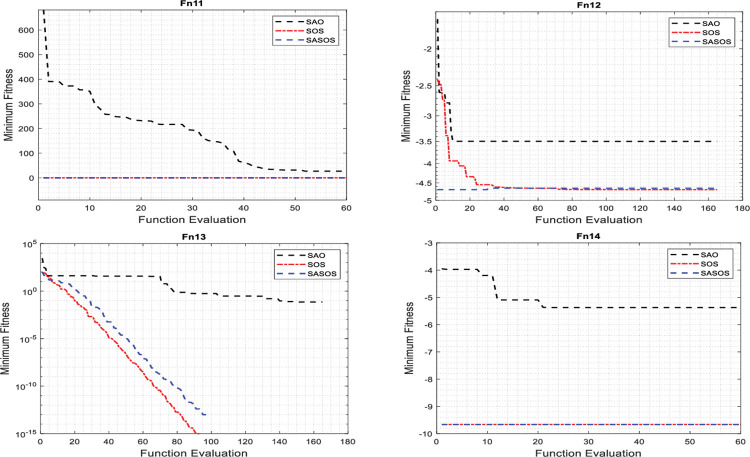
Plots of convergence curves on 4-D, 5-D and 10-D benchmark functions.

**Fig 4:**
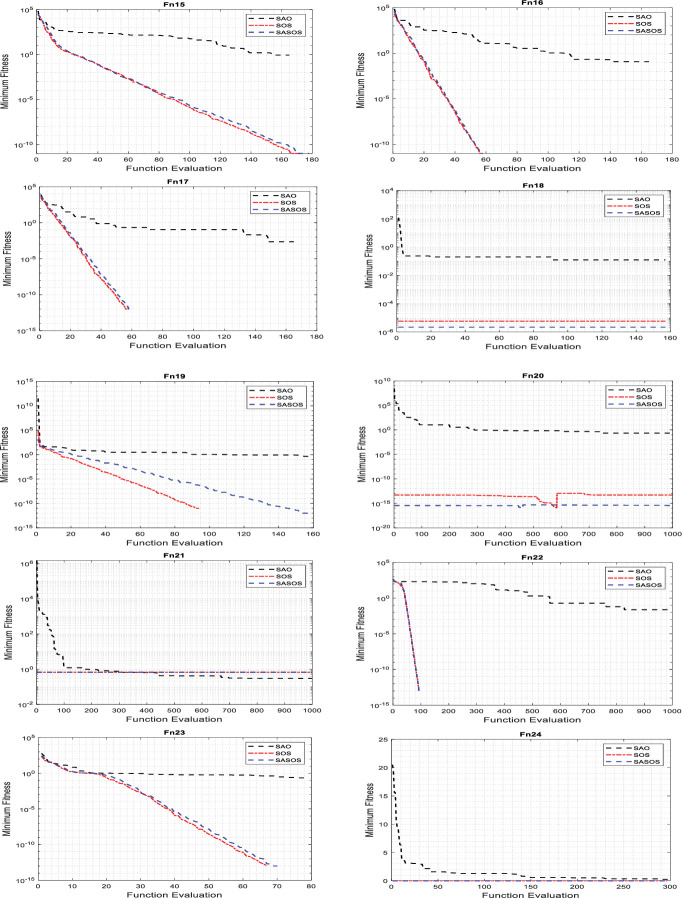
Plots of convergence curves on 30-D benchmark functions.

Convergence analyses are performed to compare the effectiveness of the fitness function between the SASOS, SOS, and SAO algorithms on benchmark functions. A reasonable number of iterations is applied in the convergence graph plot for effective performance comparison. Extended iterations allow algorithms to evaluate their fitness values on different test functions extensively. Depending on the dimensionality of each test function, some algorithms evaluate their fitness values faster to obtain an optimal solution compared to others. For a detailed comparison, we superimposed 1000 iterations on each benchmark function to plot the convergence curves as shown in Figs 2–4.

Figs 2–4 compare the convergence plots for the three algorithms in 24 benchmark functions. In the figures, the best convergence time and solution obtained simultaneously by a single algorithm in each benchmark function are considered the Desired Convergence Goal (DCG).). In the figures, it can be seen that each algorithm uniquely evaluates its minimum fitness labeled on the y-axis in the search for the best solution. The high exploitation condition in the individual algorithm can be noticed by the quick convergence against the value range of the function evaluation labeled on the x-axis. In such a condition, a solution is often obtained at high convergence speed but without a guarantee of accuracy to find the best optimal solution. In contrast, a greater exploration of the algorithm can be noticed by its wide range searchability with a higher value of the function evaluation. In this situation, the probability of obtaining the best optimal solution is high, but with a slow convergence phase that often compromises the required execution speed. However, in the DCG of an algorithm, a balance between exploitation and exploration is achieved such that the required optimal solution to the optimization problem is obtained with an insignificant imbalance between the execution speed and accuracy.

Fig 2 shows the convergence curves plotted for SASOS versus SOS and SAO algorithms on 2-D benchmark functions. These graphs show that SASOS obtained DCG in four benchmark functions (Fn1, Fn2, Fn7 and Fn9), respectively. The SOS and SAO obtained DCG each in two benchmark functions (Fn4, Fn10) and (Fn6, Fn8), respectively. On the remaining two benchmark functions (Fn3 and Fn5), no DCG was obtained by any single algorithm. Fig 3 shows the plots of convergence curves for SASOS, SOS and SAO algorithms on 4-D, 5-D and 10-D benchmark functions. In this group, SASOS obtained DCG on two benchmark functions (Fn11 (4-D) and Fn13 (10-D)). The SOS obtained DCG in Fn12 (5-D), while SAO does not have DCG in any benchmark function. On the Fn14 (10-D) benchmark function, no DCG was obtained by any algorithm. Fig 4 shows the convergence curves plotted for the SASOS, SOS, and SAO algorithms in the 30-D benchmark functions. These plots have shown that SASOS obtained DCG on four benchmark functions (Fn16, Fn17, Fn20, and Fn21). SOS did not obtain DCG in this group. However, SAO obtained DCG in two benchmark functions (Fn18 and Fn24). For the remaining four benchmark functions (Fn15, Fn19, Fn22 and Fn23), no DCG was obtained using any algorithm because the functions have negative ranges with separable values away from the global minimum. This is because their values exist in a separable range beyond the contours of the global minimum.

Of the 17 DCGs obtained by the three algorithms, SASOS obtained 10 DCGs, SOS 3 DCGs, and SAO 4 DCGs, respectively. The counts showed that SASOS leads in DCG with 58.82%, followed by SAO with 23.53% and SOS 17.65% of the total of 17 benchmark functions. It can be seen that SASOS performed better than SOS and SAO in terms of the highest number of DCGs obtained in this study. This shows that SASOS has the best search balance between the exploration and exploitation phases compared to the SAO and SOS algorithms.

### Results of microgrid control optimization

SASOS, standard SOS, and SAO algorithms are implemented in the microgrid central controller (MCC) to solve the formulated optimization problem. Synthetic per unit load is introduced into the system, and the responses of optimized control parameters are analyzed. The load model is applied as a dynamic step response having resistive, inductive, and capacitive characteristics. Change in this load induces an irregular disturbance in line impedance, flow current, supply voltage, and frequency. Optimal control of this load disturbance in steady-state operation ensures the safe operation of system equipment and improves the quality of power flow in the microgrid. The performance of the proposed optimization strategy was measured and compared with state-of-the-art optimization strategies in steady-state frequency control, voltage control, control input, and total harmonic distortion.

#### Plots of steady-state frequency control

**Fig 5:**
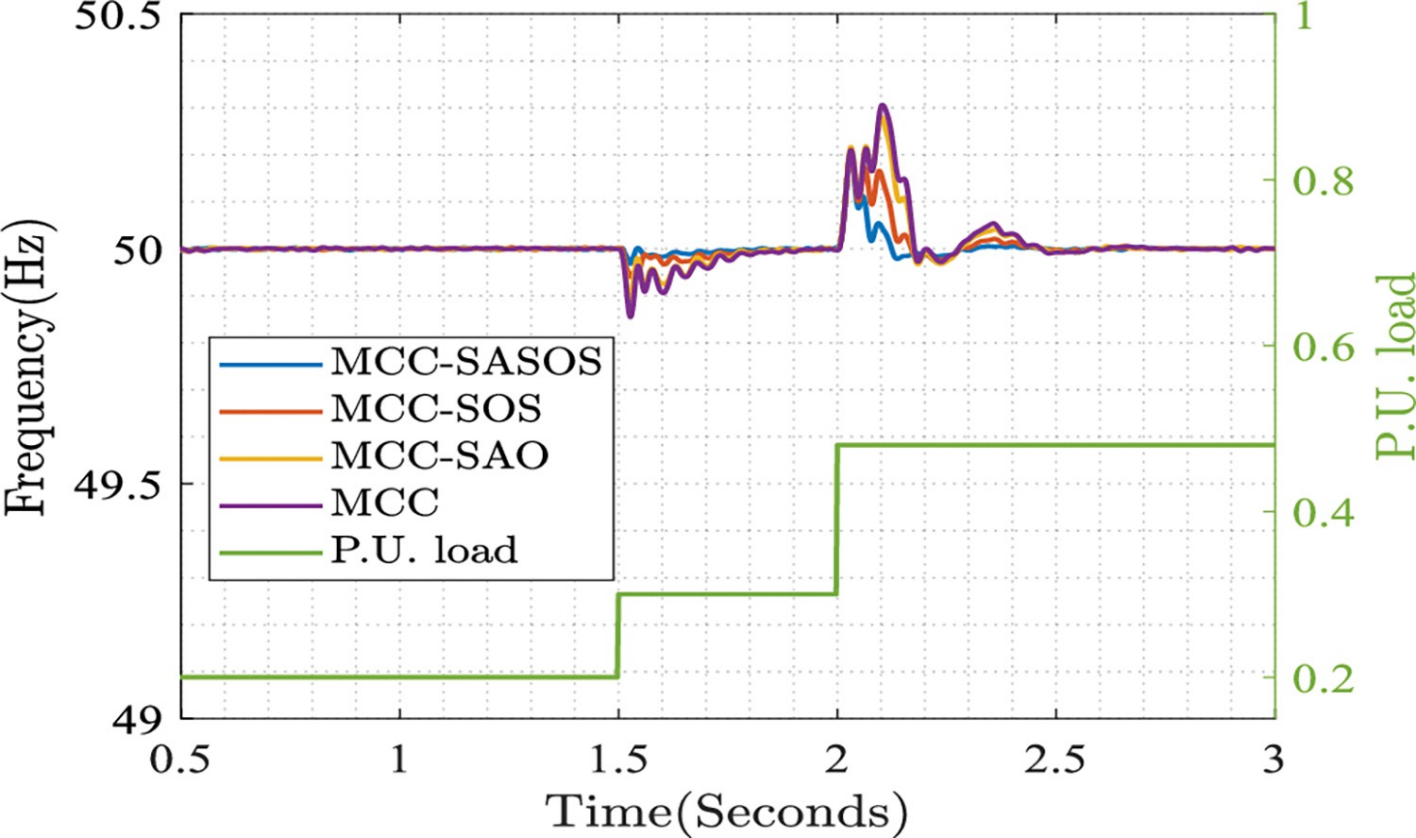
Plots of steady-state frequency control.

The plots of the steady-state frequency control in various control strategies are presented. Each strategy indicates the steady-state deviation when the unit load model is applied to the system. The applied steady-state nominal frequency control bound is 49.5≤50.0≤50.5 (HZ). The deviation in the steady state is controlled by the corresponding control strategy, as shown in Fig 5.

Fig 5 shows the steady-state frequency control in various control strategies. In the MCC-SASOS strategy plot, it can be seen that when the load increases from 0.2 to 0.3 per unit at *t* =1.50*s*, the frequency is maintained at a steady-state reference of 50HZ. As the load increases to 0.48 per unit at *t* = 20*s*, the frequency increases to 50.1HZ and stabilizes on steady-state reference at *t* = 2.15*s* onward. In the plot of the MCC-SOS strategy, the frequency decreases to 49.95HZ at *t* = 1.50*s*. When the load increases at *t* = 2.0*s*, the frequency increases to 50.15HZ and stabilizes on the reference at *t* = 2.50*s*. Similarly, in the plot of the MCC-SAO strategy, the frequency decreases to 49.90HZ at *t* = 1.50*s*, when the load increases at *t* = 2.0*s*, the frequency increases to 50.28HZ and stabilizes to reference at *t* = 2.50*s*. However, in the MCC technique plot, the frequency decreases to 49.85HZ at *t* = 1.50*s*. When the load increases at *t* = 2.0*s*, the frequency increases to 50.30HZ and stabilizes to reference at *t* = 2.50*s*. In this analysis, it can be seen that the MCC-SASOS strategy has the least frequency deviation compared to other control strategies. This showed that hybrid SASOS optimizes the control optimization problem better than the SAO and SOS algorithms.

#### Plots of steady-state voltage control

**Fig 6:**
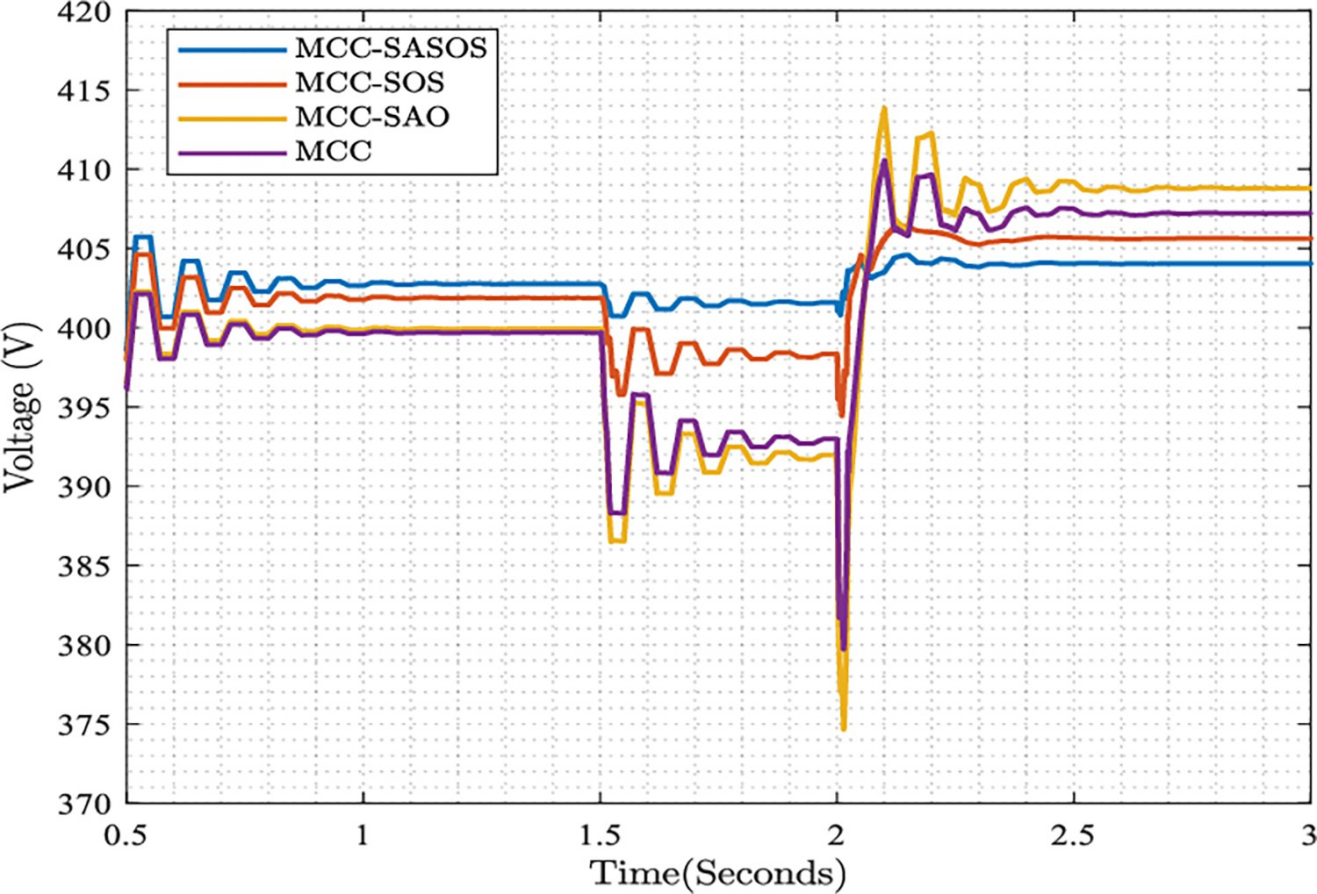
Plots of steady-state voltage control.

The plots of the steady-state voltage control using different control strategies are presented. When the per unit load applied to the system increases, the changes in steady-state voltage are controlled in each strategy. The nominal steady-state voltage supply bound is set at 380≤400≤420 (V). Fig 6 shows the control responses of various control strategies.

In the MCC-SASOS control strategy shown in Fig 6, when the load increases from 0.2 to 0.3 per unit at *t* = 1.50*s*, the steady-state supply voltage decreases from 403V to 402V. Similarly, as the load increases to 0.48 per unit at *t* = 2.0*s*, the voltage is maintained at a steady state of 404V. In the MCC-SOS strategy, the voltage drops from 402V to 395V between *t* = 1.50*s* and *t* = 2.0*s*. However, in the plots of MCC-SAO and MCC strategies, the supply voltage drops from 400V to 375V and 390V, respectively, between *t* = 1.50*s* and *t* = 2.0*s*. From these values, it can be seen from the figure that the controlled voltage in the MCC-SASOS strategy is the most stable compared to other strategies. This is because the hybrid SASOS optimizes the execution speed and accuracy of the control problem better compared to the state-of-the-art algorithms.

#### Plots of the steady-state input trajectory

**Fig 7:**
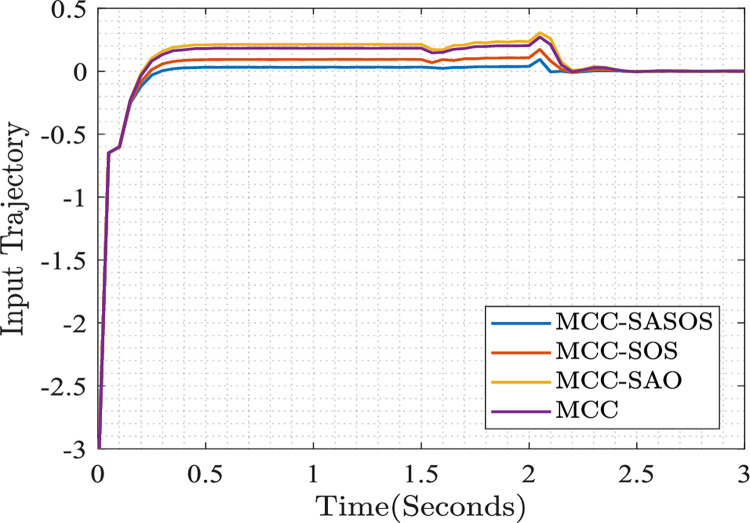
Plots of steady-state input trajectory.

The plots of steady-state input trajectories are presented for the respective control strategies. In each strategy, the control input tracks the steady-state reference. The controlled input trajectory responds to the applied per-unit load model, as shown in Fig 7.

Fig 7 shows steady-state input trajectories in different control strategies. In the plot, it can be observed that the MCC-SASOS strategy maintains the steady-state trajectory at the origin regardless of the increase in load at *t* = 1.50*s* and *t* = 2.0*s*. In the MCC-SOS strategy, the steady-state trajectory stabilizes at 0.1. As the load increases at *t* = 2.0*s*, the trajectory changes and stabilizes at the origin. However, in the MCC-SAO strategy and the MCC technique, the input trajectories stabilize at 0.2 and 0.15, respectively. When the load increases, both trajectories change to stabilize at the origin onward. It can be noted that the MCC-SASOS strategy expends the least energy in terms of steady-state input trajectory tracking compared to other control strategies.

#### Plots of total harmonics distortion

**Fig 8:**
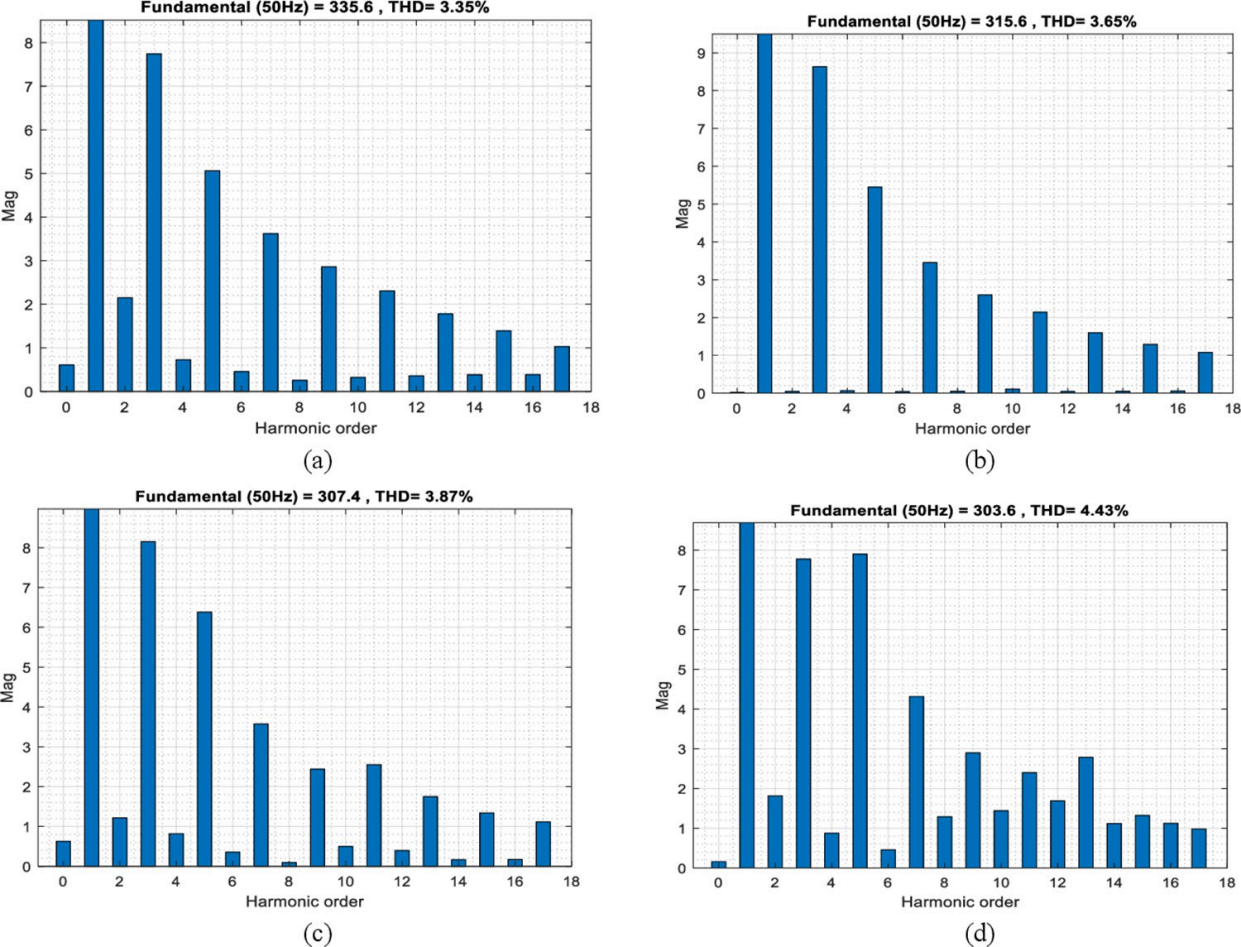
(A)—(D). Plots of Total Harmonics Distortion for Power Flow: (A) MCC-SASOS Strategy (B) MCC-SOS Strategy (C) MCC-SAO Strategy (D) MCC Technique.

The Total Harmonic Distortion (THD) plots are presented using the MCC-SASOS, MCC-SOS, MCC-SAO, and MCC strategies. Applying the load model, each strategy evaluates the odd harmonic order in the network power flow. The odd harmonic stimulates the distortion level between the signal residue and the fundamental root mean square value. A properly controlled odd harmonic shows consistent reduction order in its magnitude, and the percentage of THD is computed based on the applied sample of cycles to the fundamental frequency. For a detailed comparison, the THD graphs with respect to the fundamental frequency of 50HZ are shown in Fig 8.

Fig 8(A) shows a plot of THD for power flow using the MCC-SASOS strategy due to the applied load. It can be seen that with an increase in the harmonic order, the magnitude of the odd harmonics decreases drastically. The sample of 335.6 cycles equal to 50HZ fundamental frequency was applied to obtain a THD of 3.35%, which is 19.76% less than the recommended 5% THD benchmark. Fig 8(B) shows the THD plot using the MCC-SOS strategy. The magnitude of the odd harmonics decreases with increasing harmonic order. A sample of 315.6 cycles equal to 50HZ fundamental frequency was applied, which gives a THD of 3.65%. This resulted in a 15.60% reduction compared to the standard benchmark. Fig 8(C) shows the THD plot using the MCC-SAO strategy. In this strategy, the sample of 307.4 cycles equal to the 50HZ fundamental frequency was applied. It can be seen from the plot that the magnitude of odd harmonics decreases as the harmonic order increases, giving a THD value of 3.87%. The value is equal to a 12.97% reduction from the recommended benchmark. Fig 8(D) shows the THD value obtained using the MCC technique. The figure shows a reduction in the magnitude of odd harmonics as the harmonic order increases. The 303.6 cycles equal to the 50HZ fundamental frequency were applied to obtain a THD value of 4.43%. This gives a THD reduction of 6.04% compared to the standard 5% benchmark.

In the microgrid control optimization plots, it can be seen that in Fig 5, the MCC-SASOS control strategy obtained the lowest frequency deviation when a load was introduced into the network. This strategy’s minimum error and fastest settling time made it better compared to other control strategies. In Fig 6, it can be observed that the MCC-SASOS strategy maintained a steady-state voltage when the load increased. This strategy outperforms other compared control strategies. In Fig 7, it can be seen that the input trajectory in the MCC-SASOS strategy is more stable in steady-state and when the load is introduced. This makes the input trajectory of this strategy better in expended energy compared to other control strategies. From the THD values in Fig 8(A)–8(D), it can be seen that MCC-SASOS obtained a THD of 3.35%. The value obtained is the least, with a reduction of 19.76% over the benchmark of 5% THD. This makes the strategy better than the MCC-SOS, MCC-SAO, and MCC techniques, which obtained 3.65%, 3.87%, and 4.43% THD, showing reductions of 15.60%, 12.74%, and 6.05%, respectively, over the benchmark.

## Conclusion

In this paper, a hybrid smell agent Smell Agent Symbiosis Organism Search Algorithm (SASOS) algorithm for the optimization of autonomous microgrid control has been implemented. Due to the varying load demand, various models of the energy resources were coordinated into a unit model to achieve optimal energy generation and delivery to active loads. The optimization problem is formulated based on the power flow of the model and the discrete-time sampling of the constrained control parameters. For an effective solution to the optimization problem, SASOS is codified using the fundamental components of SOS and SAO in a recurring optimization loop. SASOS was evaluated in 24 selected test functions and compared with the SOS and SAO algorithms. The performance of each algorithm was measured using the counts of Desired Convergence Goals (DCG) in the benchmark functions. Experimental analysis revealed that SASOS obtained 58.82% DCG in 17 of the benchmark functions, followed by SAO and SOS with 23.53% and 17.65% DCG, respectively. The algorithms were implemented in the Microgrid Central Controller (MCC) in the respective control strategies. When a synthetic unit load was applied to each strategy, steady-state deviations in frequency, voltage, input trajectory, and Total Harmonic Distortion (THD) were evaluated. Simulation results in MATLAB/Simulink R2020b showed that the MCC-SASOS strategy achieved better stability in the control parameters with a reduced THD of 19.76% compared to the SOS, SAO and MCC methods having reductions of 15.60%, 12.74% and 6.04%, respectively, over the standard THD benchmark.

In future work, SASOS is recommended to be used in solving other segments of microgrid optimizations, such as optimal placement of distributed generators, sizing optimization of microgrid components, power loss minimization and control optimization of stochastic load disturbances. Similarly, the algorithm can be applied to several areas of engineering practice, such as lane detection and navigation accuracy optimization, image processing enhancement, optimal active slosh control, task scheduling, path planning, and several applications.
